# Yiqi Wenyang decoction protects against the development of atherosclerosis by inhibiting vascular inflammation

**DOI:** 10.1080/13880209.2025.2492650

**Published:** 2025-04-20

**Authors:** Shuang Hua, Lingling Sun, Han Zhang, Chiwen Shiu, Shujie Zhang, Yao Zhu, Xingqun Yan, Ping Gu, Zhe Huang, Weimin Jiang

**Affiliations:** a Department of Cardiology, Affiliated Hospital of Nanjing University of Chinese Medicine, Nanjing, China; b Department of Cardiology, The Second Affiliated Hospital of Zhejiang Chinese Medical University, Zhejiang, China; c Department of Endocrinology, Jinling Hospital, School of Medicine, Nanjing University, Nanjing, China; d Department of Endocrinology, Jinling Hospital, the First School of Clinical Medicine, Southern Medical University, Nanjing, China; e Department of Genetics and Developmental Science, School of Life Sciences and Biotechnology, Shanghai Jiao Tong University, Shanghai, China; f Sheng Yushou Center of Cell Biology and Immunology, School of Life Sciences and Biotechnology, Shanghai Jiao Tong University, Shanghai, China; g Department of Cardiology, Shanghai Pudong New Area People’s Hospital, Shanghai, China

**Keywords:** Atherosclerosis, Yiqi Wenyang decoction, vascular inflammation, NF-κB

## Abstract

**Context:**

Vascular inflammation is a key process in the pathogenesis of atherosclerosis, which is regulated by NF-κB pathway. Yiqi Wenyang decoction (YQWY), a Traditional Chinese medicine (TCM) formula, has anti-inflammatory properties and may inhibit this pathway, potentially offering anti-atherosclerotic effects.

**Objective:**

The purpose of this study is to investigate the effects of YQWY on atherosclerosis and the underlying mechanism. Materials and methods: ApoE^−/−^ mice were fed a Western diet and administered with YQWY (low or high dose), atorvastatin, or vehicle for 13 weeks. The size of atherosclerotic plaques was assessed using ORO staining. Vascular inflammation was evaluated with IF or IHC staining. The mechanisms and signaling pathways underlying the effect of YQWY on vasculature were studied using transcriptomic analysis and were validated *in vitro* in endothelial cells and macrophages.

**Results:**

YQWY attenuated atherosclerotic plaque development which was associated with reduced vascular inflammation as demonstrated by transcriptomic analysis of aorta. This was verified by reduced expression of proinflammatory chemokines, adhesion molecules, and inflammatory cytokines in aortas from YQWY-treated mice at both mRNA and protein levels. Mechanistically, YQWY suppressed NF-κB activation in endothelial cells and, to a lesser extent, macrophages possibly.

**Discussion and conclusions:**

YQWY protects against vascular inflammation and atherosclerosis by suppressing NF-κB pathway, suggesting the potential of YQWY and its active ingredients as novel anti-atherosclerotic therapeutics.

## Introduction

Atherosclerosis is a chronic inflammatory disease of the arteries that can lead to stroke, peripheral vascular disease, and ischemic heart disease, collectively known as cardiovascular diseases (CVDs) (Kobiyama and Ley 2018). Endothelial cell dysfunction resulting from endothelial activation is recognized as one of the initial events in the pathogenesis of atherosclerosis (Sun et al. 2019). In activated endothelial cells, the expression of chemokines, such as C–X–C motif chemokine ligand 1 (CXCL1), C–C motif chemokine ligand 5 (CCL5), and adhesion molecules, such as vascular cell adhesion molecule 1 (VCAM-1) and intercellular adhesion molecule 1 (ICAM-1) is significantly upregulated, leading to the recruitment, tethering, and stable arrest of circulating immune cells, especially monocytes, along the endothelial monolayer of the arterial wall and subsequent migration to the intima (Sun et al. 2019). After infiltration into the intima, monocytes differentiate into macrophages, which absorb excessive lipoproteins and are transformed into cholesterol-laden foam cells, thereby inducing nascent plaque formation (Gisterå and Hansson 2017).

Nuclear factor kappa-B (NF-κB) also contributes to vascular inflammation in atherosclerotic plaque formation. NF-κB is comprised of five structurally related members, namely NF-κB1 (p50), NF-κB2 (p52), c-Rel, RelA (p65), and RelB forming homo- or hetero-dimers (Liu et al. 2017). NF-κB is usually sequestered in cytoplasm by inhibitory proteins, including IκB family proteins. Canonical NF-κB activation occurs through site-specific phosphorylation and degradation of IκB mediated by IκB kinase, which results in the release of NF-κB and the subsequent nuclear translocation (Liu et al. 2017). IκB kinases are activated in response to various stimuli, including tumor necrosis factor-α (TNF-α), interleukin-1 (IL-1), and lipopolysaccharides (LPS) (Lee and Hung 2008). NF-κB activation leads to the transcription of target genes encoding cytokines, chemokines, and adhesion molecules in different cells (Liu et al. 2017). Consequently, aberrant activation of NF-κB signaling is a hallmark of vascular inflammation in the development and progression of atherosclerosis (Kong et al. 2022). Therefore, anti-inflammatory therapeutics targeting NF-κB signaling present a promising strategy for treating atherosclerosis.

Extensive experimental and clinical evidence has proven the effectiveness of traditional Chinese medicine (TCM) in treating CVDs, including atherosclerosis, hypertension, and chronic heart failure (Yang et al. 2023). Yiqi Wenyang (YQWY) has been used as an in-hospital TCM medicine preparation at Jiangsu Provincial Hospital of Traditional Chinese Medicine for the treatment of chronic heart failure with remarkable efficacy for decades (Huang et al. 2019). YQWY is a TCM formula made from Huangqi (*Astragalus membranaceus* (Fisch.) Bge., Fabaceae), Hongjingtian (*Rhodiola rosea* L., Crassulaceae), Fuzi (*Aconitum carmichaelii* Debeaux, Ranunculaceae), Zhuling (*Polyporus umbellatus* (Pers.) Fr., Polyporaceae), Tinglizi (*Lepidium apetalum* Willd., Brassicaceae), Ezhu (*Curcuma phaeocaulis* Valeton, Zingiberaceae), Baishao (*Paeonia lactiflora* Pall., Ranunculaceae), and Shengjiang (*Zingiber officinale* Rose., Zingiberaceae). YQWY has been shown that improve myocardial remodeling by reducing inflammation, fibrosis, and apoptosis in the myocardium through inhibition of NF-κB signaling (Li et al. 2020). In addition to the treatment of heart failure, YQWY has been observed to yield effective clinical outcomes in treating atherosclerosis at Jiangsu Provincial Hospital of Traditional Chinese Medicine. However, the mechanism by which YQWY treats atherosclerosis remains unclear.

Given that NF-κB activation in endothelial cells and macrophages induces vascular inflammation and promotes the development of atherosclerosis, as reported in various studies (Zimmer et al. 2015; Kong et al. 2022), this study aims to assess the effectiveness of YQWY in mitigating atherosclerosis using a mouse model of atherosclerosis and to investigate the underlying molecular mechanism using *in vitro* experiments. Here, we elucidated an anti-atherosclerotic role of YQWY by inhibiting NF-κB activation in endothelial cells and, to a lesser extent, in macrophages.

## Materials and methods

### Preparation of YQWY

All botanical drugs were sourced from the Pharmacy Department of Jiangsu Province Hospital of TCM (Nanjing, China). The YQWY prescription comprises eight Chinese botanical drugs, namely Huangqi (*Astragalus membranaceus* (Fisch.) Bge., Fabaceae, 60 g), Hongjingtian (*Rhodiola rosea* L., Crassulaceae, 15 g), Fuzi (*Aconitum carmichaelii* Debeaux, Ranunculaceae, 10 g), Zhuling (*Polyporus umbellatus* (Pers.) Fr., Polyporaceae, 15 g), Tinglizi (*Lepidium apetalum* Willd., Brassicaceae, 15 g), Ezhu (*Curcuma phaeocaulis* Valeton, Zingiberaceae, 10 g), Baishao (*Paeonia lactiflora* Pall., Ranunculaceae, 12 g), and Shengjiang (*Zingiber officinale* Rose., Zingiberaceae, 6 g). These botanical drugs were soaked in ten times their volume of water for 1.5 h, followed by decoction for 45 min. Subsequently, they were concentrated and extracted into lyophilized powder (yield: 19%), stored at −80 °C, and diluted with sterile pure water before use. The chemical analysis of YQWY was detected using UPLC/LTQ-Orbitrap-MS, with the total ion chromatogram and specific methods in Supplemental Figure 1, and identified chemical components in Supplemental Table 1.

### Animals and treatment

All animal experimental procedures were performed following the NIH’s Guide for the Care and Use of Laboratory Animals and approved by the Animal Care and Protection Committee of Nanjing University of Chinese Medicine (No. ACU230201). C57BL/6J and ApoE^−/−^ mice (male, 8-week-old) were purchased from SiPeiFu Biotechnology Co., Ltd (Beijing, China). The mice were maintained under specific pathogen-free (SPF) conditions at Nanjing University of Chinese Medicine and were subsequently divided into five groups: a normal control group (NC), consisting of C56BL/6J mice fed with standard chow diet (STD) for 13 weeks; an atherosclerosis model group, consisting of ApoE^−/−^ mice fed with Western diet (WD) containing 40 kcal% fat and 0.15% cholesterol for 13 weeks; and four treatment groups. Based on the ‘Practice Guide for Dose Conversion Between Animals and Humans’ (Nair and Jacob 2016), a dose scalability factor of 12.3 is often used to convert human doses to mouse equivalent doses. Therefore, the clinical equivalent gavage dose concentration for mice is approximately 0.55 g/mL, administered in 0.1 mL/10 g doses once daily by gavage. The atherosclerotic mice were divided into four groups receiving daily treatment *via* oral gavage: a model group receiving sterile water; a YQWY low-dose group (YQWY-L) receiving YQWY at the clinically equivalent dose (0.55 g/mL); a YQWY high-dose group (YQWY-H) receiving twice the clinically equivalent dose of YQWY (1.1 g/mL); and an atorvastatin group (AT) receiving atorvastatin at 6 mg/kg. After the treatment period, the mice were deeply anesthetized with avertin and subsequently euthanized. Serum, liver, and aorta were collected for further analysis.

### Quantification of atherosclerotic plaques and immunostaining

Mouse aortic root sections and the entire aorta trunk were fixed in 4% paraformaldehyde overnight and stained by oil Red O [30% (w/v)] (Sigma-Aldrich, 01391) for 15 min followed by washing with 60% isopropyl alcohol for 1 min. Immunofluorescence (IF) staining was performed in aortic root sections of mice with antibodies against VCAM-1 and CD68 overnight at 4 °C. The sections were then washed and incubated for 1 h at room temperature in the dark with fluorescently conjugated secondary antibodies. Slides were counterstained with DAPI (Servicebio, G101). For immunohistochemistry (IHC) staining, deparaffinized sections were incubated with the following primary antibodies: IL-1β, ICAM-1, TNF-α, and CXCL1 in phosphate-buffered saline (PBS) containing 3% bovine serum albumin overnight at 4 °C, followed by incubation with HRP-conjugated secondary antibodies and developed by DAB (Servicebio, #G1212). Subsequently, nuclei were counterstained with hematoxylin (Servicebio, #G1004). Tissue sections were visualized with a panoramic scanning system (Panoramic MIDI, 3D HISTECH). The signal-positive area was outlined and quantified by ImageJ software.

### HE and Masson trichrome staining

Mouse aortic root and liver sections were fixed with 4% PFA and stained with H&E (Servicebio, G1005) for histological analysis or with Masson trichrome (Servicebio, G1006) for the analysis of collagen fibers.

### Biochemical and immunological assays

12-h fasted plasma lipid profiles and liver function including total triglycerides (TG, #A110-1-1), total cholesterol (TC, #A111-1-1), low-density lipoprotein (LDL, #A113-1-1), high-density lipoprotein (HDL, #A112-1-1), alanine transaminase (ALT, #C009-2-1), aspartate aminotransferase (AST, #C010-2-1), and total bile acids (TBA, #E003-2-1) were measured with commercial kits from Nanjing Jiancheng Bioengineering Institute. Serum chemotactic and proinflammatory factors, including CXCL1 (#AF2810-A), IL-1β (#AF2040-A), and TNF-α (#AF2132-A) (AiFang Biological, China), were measured using a double-antibody one-step sandwich ELISA kit according to the manufacturer’s instructions.

### Cell culture and treatment

The human endothelial hybrid cell line cell (EA.hy926) and mouse leukemic monocyte/macrophage cell line (RAW264.7) were obtained from the Cell Bank of Typical Culture Preservation Committee of Chinese Academy of Sciences (Shanghai, China), and cultured in high-glucose DMEM containing 10% FBS (Hyclone, #SV30208) and 1% penicillin/streptomycin (Gibco, #15140122). Lyophilized YQWY powder was dissolved in PBS and sterile filtered before being added to cells at the given concentrations. EA.hy926 and RAW264.7 cells were pretreated with YQWY (100 or 200 μg/mL) for 6 h, and subsequently treated with 20 ng/mL recombinant human TNF-α (R&D Systems, #210-TA-020), or 1 μg/mL LPS from E. coli 0111:B4 (Sigma–Aldrich, #L4391) for different durations as indicated. Subsequently, cells were collected for protein and RNA extraction.

### Cytological staining

EA.hy926 and RAW264.7 cells were pretreated with YQWY (100 or 200 μg/mL) for 6 h and then stimulated with recombinant human TNF-α or LPS for 30 min, respectively. The cells were then incubated with the primary antibody (NF-κB p65) overnight at 4 °C. Subsequently, fluorescently conjugated secondary antibodies were added and incubated for 1 h at room temperature before adding DAPI. The semi-quantitative analysis of nuclear translocation was performed using the ImageJ plugin-Colocalization Finder based on Manders’ coefficients (Di Tomaso et al. 2022). Images were acquired using an upright and inverted integrated fluorescence microscope (Echo Revolve, R4).

### Cell viability assay

EA.hy926 and Raw 264.7 cells were seeded in 96-well plates at densities of 1 × 10^4^ and 2 × 10^4^ cells per well, respectively. After culturing to 80% confluence, the cells were treated with various doses of YQWY for 24 h. Cell viability was assessed using the Cell Counting Kit-8 (CCK-8) (Meilun, #MA0218), and viability was quantified as the percentage of absorbance in the experimental groups relative to the untreated control group.

### RNA extraction and RT–qPCR analysis

Total RNA was extracted from tissues or cells using RNAiso Plus (Takara, #9109), followed by cDNA synthesis using a reverse transcription reaction reagent (Takara, #RR037A). RT-qPCR analysis was conducted utilizing the ChamQ Universal SYBR qPCR master mix (Vazyme, #Q711-02) on a qTOWER3G Real-Time PCR System (Analytik-Jena, Germany). The expression levels of all target genes were normalized to the housekeeping gene β-actin, and relative quantification was performed using the ΔCt method. Primer sequences are listed in Supplemental Table 2.

### RNA-seq and data analysis

At the end of the animal study, aortas were harvested from mice in the control, model, and YQWY treatment groups. Total RNA was isolated from the samples using RNAiso Plus (Takara, #9109). The quality and integrity of purified RNA were examined with 2100 Bioanalyser (Agilent). Sequencing libraries were generated using TruSeqTM RNA sample preparation Kits from Illumina (San Diego, CA) according to the manufacturer’s protocol. Paired-end sequencing was performed at Majorbio Bio-Pharm Technology Co. Ltd. (Shanghai, China) with a sequencing per million reads. RNA sequencing raw data were analyzed using SeqPrep (https://github.com/jstjohn/SeqPrep) and Sickle (https://github.com/najoshi/sickle) for mapping reads to a reference mouse genome, quantification, and differential expression analysis using the RSEM (http://deweylab.biostat.wisc.edu/rsem/) and DESeq2/DEGseq/EdgeR. FC ≥ 1.2 and *p* value < 0.05 were used as the cut-off point for the identification of differentially expressed genes (DEGs).

### Western blot analysis

Proteins were isolated using cell lysis buffer (Beyotime Biotechnology, #P0013J) supplemented with protease and phosphatase inhibitor cocktail (Beyotime Biotechnology, #P1050). 25 μg of protein was loaded into each well of a 10% SDS-page gel (EpiZyme Biotechnology, PG112). The membrane was blocked with 5% BSA, incubated with primary antibodies overnight at 4 °C, and then with secondary antibodies for 1 h at room temperature. Blots were visualized using a chemiluminescence imager (Tanon, #4600) and an ECL kit (EpiZyme Biotechnology, #SQ202L). For the same membrane, the phosphorylated antibodies were first incubated, and then the previously bound primary and secondary antibodies were stripped from the PVDF membrane. The following steps are the same as above. Comparative densitometry of the bands was performed using ImageJ software and normalized to the density of the corresponding GAPDH.

### Antibody

The antibodies used in the IHC, IF, and WB are listed in Supplemental Table 3.

### Statistics

Data are shown as mean ± SD. GraphPad Prism software (V.9.5.1) was used for data analysis. Statistical differences were calculated using one-way ANOVA with Tukey’s post hoc test. *P* values less than 0.05 were considered statistically significant, and *P* values less than 0.01 were considered statistically highly significant.

## Results

### YQWY alleviates Western diet-induced atherosclerosis in ApoE^−/−^ mice

**Figure 1.**
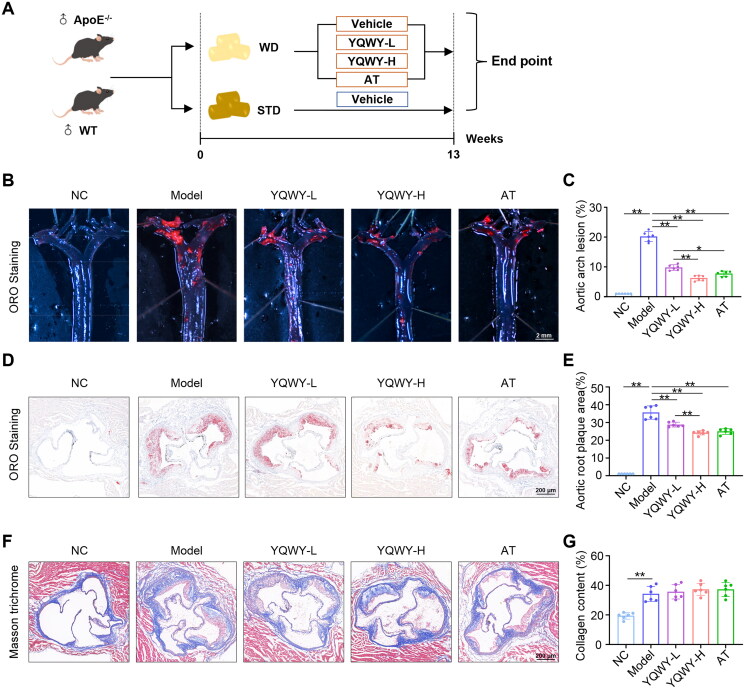
YQWY attenuates atherosclerosis in WD-fed ApoE^−/−^ mice. 8-week-old male ApoE^−/−^ mice were fed with Western diet (WD) and treated with vehicle, YQWY at 0.55 or 1.1 g/mL/d, or atorvastatin at 6 mg/kg/d as the model, low-dose YQWY (YQWY-L), high-dose YQWY (YQWY-H), and AT groups, respectively for 13 weeks. C57BL/6J mice fed with standard chow diet (STD) and treated with sterile water were used as the normal control (NC) group. (A) Schematic diagram of experimental animal model and treatment regimen. Representative images of oil Red O (ORO) staining of the aortic arch (B) and aortic root plaque (D), and masson trichrome stained aortic roots reveal collagen deposition (F). The fractional plaque area in the total aorta arch (C), plaque area in the aortic root (E), and collagen content in aortic root (G) (*n* = 6). Data are shown as mean ± SD. *p < 0.05, **p < 0.01. Scale bar: *B* = 2 mm; D, *F* = 200 μm.

To examine the impact of YQWY on atherosclerosis, ApoE^−/−^ mice were fed a WD for 13 weeks to induce atherosclerosis (Figure 1(A)). Analysis *via* ORO staining of the entire aorta and cross-sections of the aortic sinus revealed significant plaque formation in the aortic arch and lipid deposition in the aortic sinus within the model group, but not in the NC group, confirming the successful establishment of atherosclerosis in the model group. Compared with the model group, YQWY treatment significantly reduced plaque formation in both aortic arch and aortic root in a dose-dependent manner comparable to the effects observed with atorvastatin, a statin medication used to treat dyslipidemia and prevent CVDs (Figure 1(B–E)). Additionally, YQWY did not significantly increase collagen deposition in the aortic root (Figure 1(F–G)). Since lipid dysregulation is a major contributor to the initiation and progression of atherosclerosis, we carefully measured serum lipid levels and hepatic lipid accumulation. Our findings revealed that only serum HDL level was mildly elevated in mice treated with YQWY, while serum levels of TG, TC, LDL, and hepatic lipid content were not significantly affected (Fig. S2A–E). Furthermore, considering the potential hepatotoxicity associated with excessive consumption of botanical drugs, we also tested the effect of YQWY on liver injury. Mice fed with 13-week WD exhibited liver injury as reflected by high serum levels of ALT, AST, and TBA compared to the NC group. Importantly, YQWY treatment did not further increase these serum markers of liver injury (Fig. S3A–C). Histological analysis of liver sections through hematoxylin and eosin staining revealed that YQWY did not induce hepatic lobular inflammation (Fig. S3D). These findings indicate that YQWY treatment effectively protects against atherosclerosis development independent of lipid-lowering effects.

### YQWY inhibits inflammatory pathways in the vasculature

**Figure 2.**
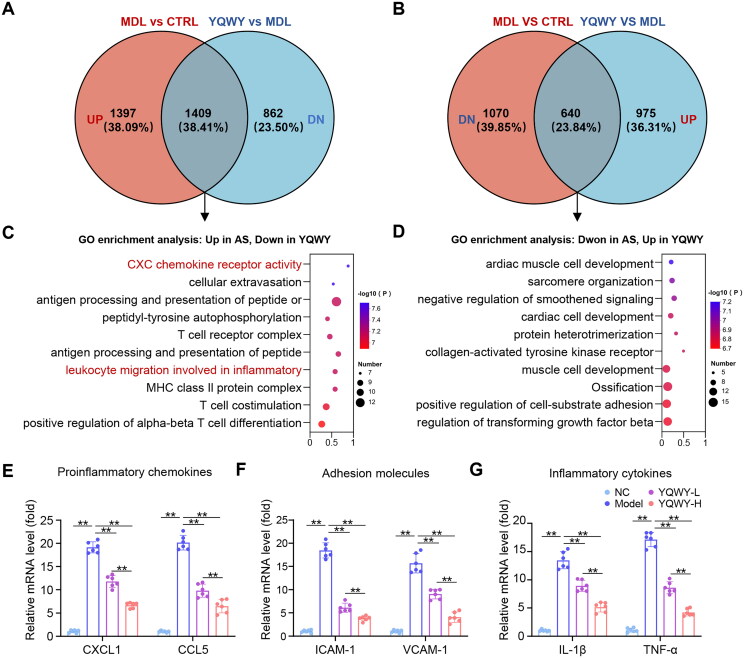
YQWY suppresses proinflammatory pathways in aorta from WD-fed ApoE^−/−^ mice. 8-week male C57BL/6J mice on STD and ApoE^−/−^ mice on WD were treated with respective vehicles, YQWY 0.55 or 1.1 g/mL/d for 13 weeks. RNA-sequencing data were acquired from aortic specimens to identify differentially expressed genes (DEGs). (A) Venn diagram analysis of gene or transcript expression between the up-regulated (up) DEGs among model (MDL) group and control (CTRL) group against the down-regulated (DN) DEGs between YQWY treatment (YQWY) group and MDL groups (*n* = 3, FC ≥ 1.2; *p* < 0.05). (B) In contrast, Venn diagram analysis of gene or transcript expression between the DN-DEGs among the MDL group and CTRL group against the up-DEGs between YQWY group and MDL groups (*n* = 3, FC ≥ 1.2; *p* < 0.05). (C–D) GO analysis of DEGs in the intersection gene was obtained from the a and B diagrams, respectively. RT-qPCR analysis of proinflammatory chemokines (E), adhesion molecules (F), and inflammatory cytokines (G) in aortas (*n* = 6). β-actin was used as the reference gene. Data are shown as mean ± SD. *p < 0.05, **p < 0.01.

To gain insights into the mechanism underlying the anti-atherosclerotic effects of YQWY, transcriptomic analysis of intact aorta samples obtained from the control (CTRL), model (MDL), and YQWY treatment (YQWY) groups were performed. Compared to the CTRL group, the MDL group had 2806 genes significantly upregulated (1397 + 1409, Figure 2(A), orange and intersecting area) and 1710 genes significantly downregulated (1070 + 640, Figure 2(B), orange and intersecting area). When comparing the YQWY treatment group to the MDL, 2271 genes were significantly downregulated (1409 + 862, Figure 2(A), blue and intersecting area), while 1615 genes were significantly upregulated (640 + 975, Figure 2(B), blue and intersecting area) (*p* < 0.05, FC ≥ 1.2). To further identify the differentially expressed genes (DEGs) suppressed by YQWY that may be involved in atherosclerosis, we overlapped the DEGs identified in the MDL vs CTRL and YQWY vs MDL. Among the DEGs between the MDL vs CTRL, YQWY treatment significantly downregulated 1409 genes that were up-regulated in the MDL group (Figure 2(A), intersecting area) and upregulated 640 genes that were down-regulated in the MDL group (Figure 2(B), intersecting area). Notably, unbiased Gene Ontology (GO) enrichment analysis revealed that the genes upregulated in atherosclerosis and downregulated by YQWY treatment were significantly enriched for the chemokine receptor activity, leukocyte-mediated inflammation, and other lymphocyte activation pathways Conversely, the genes downregulated in atherosclerosis and upregulated by YQWY treatment were significantly enriched for the transforming growth factor receptors beta-related pathway, which may promote plaque stability (Toma and McCaffrey 2012) (Figure 2(C and D)). These findings were further validated by RT-qPCR analysis. Aortic tissue of mice fed a WD exhibited significantly higher expression levels of genes associated with proinflammatory chemokines (CXCL1 and CCL5), adhesion molecules (VCAM-1 and ICAM-1), and inflammatory cytokines (IL-1β and TNF-α) compared to the NC group (Figure 2(E–G)). Importantly, YQWY treatment significantly attenuated these genes in a dose-dependent manner (Figure 2(E–G)). Collectively, these data suggest that YQWY inhibits atherosclerosis-induced vascular inflammatory responses.

### Mice treated with YQWY demonstrate reduced inflammatory cell infiltration in the aorta

**Figure 3.**
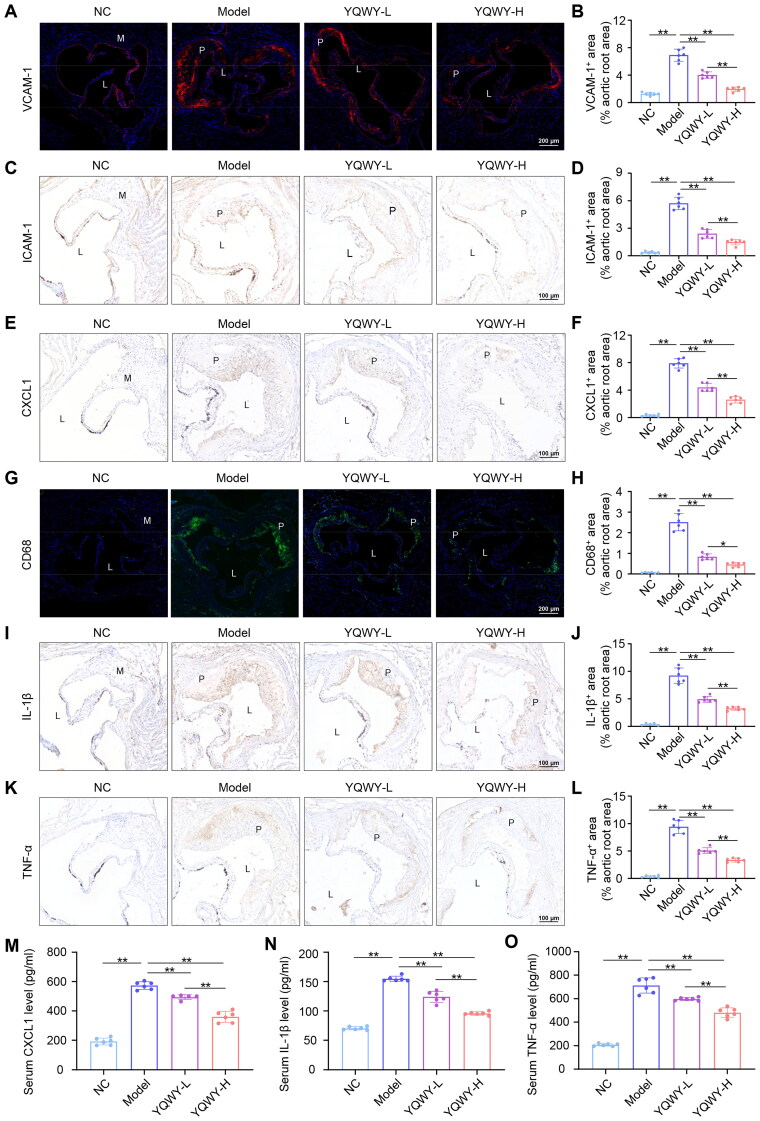
YQWY-treated mice exhibit reduced infiltration of inflammatory cells in aorta and lower level of systemic inflammation. 8-week male C57BL/6J mice with STD and ApoE^−/−^ mice with WD were dosed with vehicle, or YQWY for 13 weeks. Representative immunofluorescence (IF) staining images of (A) VCAM-1 (red) and (G) CD68 (green) in aortic roots, and the related quantitative analysis, respectively (B, H, *n* = 6). Blue: DAPI. Representative immunohistochemical staining images of (C) ICAM-1, (E) CXCL1 (I) IL-1β, (K) TNF-α, in aortic roots, and the related quantitative analysis, respectively (D, F, J, L, *n* = 6). (M–O) Serum CXCL1, IL-1β, and TNF-α were detected by ELISA, respectively (*n* = 6). Data are shown as mean ± SD. *p < 0.05, **p < 0.01. Scale bar: A, *G* = 200 mm; C, E , I, *K* = 100 μm.

To substantiate the anti-inflammatory effects of YQWY in the vasculature, we conducted IF and IHC staining on the aortic root. It was shown that the number of cells expressing adhesion molecules (VCAM-1 and ICAM-1) and chemokines (CXCL1) positive cells, indicative of activated endothelial cells, and CD68 positive cells, indicative of macrophages, were significantly increased in the aortic roots of mice fed with WD for 13 weeks (Figure 3(A–H)). Notably, treatment with YQWY markedly reduced the abundance of these cells in atherosclerotic mice in a dose-dependent manner (Figure 3(A–H)). Moreover, YQWY treatment dose-dependently reduced the positive area for proinflammatory cytokines (IL-1β and TNF-α) in aortic plaques (Figure 3(I–L)). In line with these results, administration with YQWY led to a mild but significant reduction in circulating levels of these chemokine and proinflammatory cytokines in mice with atherosclerosis (Figure 3(M–O)). Collectively, these findings indicate that YQWY demonstrates anti-atherosclerotic properties by suppressing the infiltration of inflammatory cells into atherosclerotic lesions.

### YQWY suppresses endothelial cell activation and macrophage inflammation

**Figure 4.**
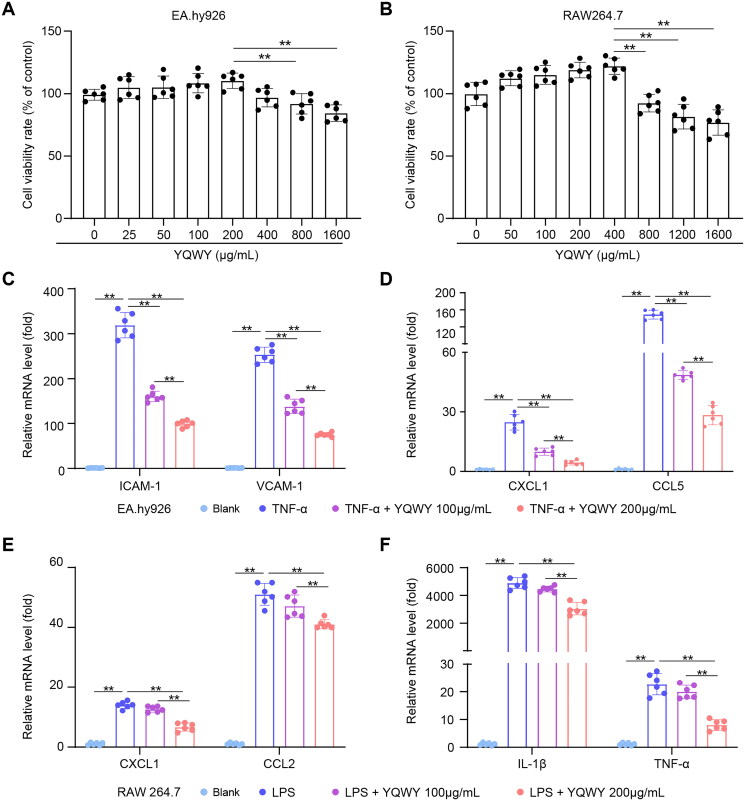
YQWY inhibits the expression of chemokines and adhesion molecules in endothelial cells and the expression of proinflammatory chemokines and cytokines in macrophages. EA.hy926 cells were stimulated with TNF-α (20 ng/mL) and YQWY (100 or 200 μg/mL), and RAW264.7 cells were stimulated with LPS (1 μg/mL) and YQWY (100 or 200 μg/mL) for different times, according to the experiment: CCK-8, 24h, RT-qPCR, 6 h. (A-B) EA.hy926 and RAW264.7 cells were treated with YQWY in a dose-dependent manner for 24 h, and CCK-8 assay was conducted to detect cell viability. (C) Relative expression of adhesion molecules (ICAM-1 and VCAM-1), and (D) proinflammatory chemokines (CXCL1 and CCL5) mRNA in EA.hy926 cells (*n* = 6). (E) Relative expression of proinflammatory chemokines (CXCL1 and CCL2), and (F) inflammatory cytokines (IL-1β and TNF-α) mRNA in RAW264.7 cells (*n* = 6). Data are shown as mean ± SD. *p < 0.05, **p < 0.01.

To further dissect the effect of YQWY on vascular inflammation, we investigated its impact on endothelial cells (EA.hy926) and macrophages (RAW264.7) *in vitro*. First, we used the CCK-8 cell viability/cytotoxicity assay to determine the safe concentration range of YQWY for both cell lines. The results showed that YQWY was cytotoxic to EA.hy926 cells and RAW264.7 cells at concentrations exceeding 200 and 400 μg/mL, respectively (Figure 4(A and B)). Therefore, 100 μg/mL and 200 μg/mL of YQWY were selected as the highest non-cytotoxic concentrations for subsequent *in vitro* studies.

Next, we employed well-established cellular models of inflammation: TNF-α was used to treat EA.hy926 cells to induce endothelial activation and LPS was used to treat RAW264.7 cells to induce macrophage inflammation. These stimuli are commonly used in cellular experiments to mimic vascular inflammation observed in atherosclerosis (Simion et al. 2020; Liu et al. 2023). Our results indicated that YQWY dose-dependent decreased the expression of adhesion molecules and chemokines in endothelial cells (Figure 4(C and D)). Conversely, only high doses of YQWY were effective in reducing the expression of proinflammatory cytokines and chemokines in macrophages (Figure 4(E and F)), suggesting a lower responsiveness of macrophages to YQWY compared to endothelial cells.

### YQWY inhibits the NF-κB signaling pathway in vivo and in vitro

**Figure 5.**
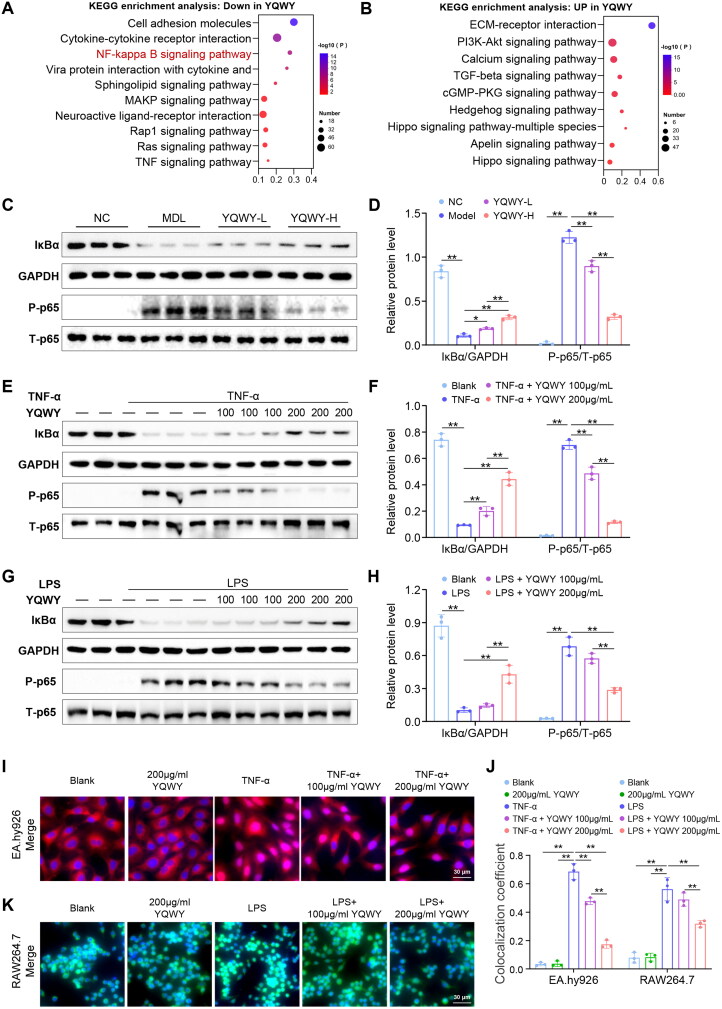
YQWY inhibits NF-κB signaling pathway *in vivo* and *in vitro*. YQWY and vehicle were administered independently to the 8-week male ApoE^-/-^ mice for 13 weeks. KEGG enrichments of (A) Down-DEGs and (B) UP-DEGs from mouse aortas between the YQWY treatment and model groups (*n* = 3, FC ≥ 1.2; *p* < 0.05). (C) Representative immunoblot images and (D) quantitative data of IκBα and P-p65 in mouse aorta, respectively. The EA.hy926 cells were stimulated with TNF-α (20 ng/mL) and YQWY (100 or 200 μg/mL), and RAW264.7 cells were stimulated with LPS (1 μg/mL) and YQWY (100 or 200 μg/mL) for 30 min. (E, G) Representative immunoblot images and (F, H) quantitative data of IκBα and P-p65 in EA.hy926 and RAW264.7 cells, respectively. Quantitative data representing the fold change of phosphorylated proteins were normalized against their respective total protein, and GAPDH was used as the loading control (*n* = 3). (I, K) Representative images of NF-κB/p65 nuclear translocation in EA.hy926 (red) and RAW264.7 (green) cells were visualized by IF staining and nuclei stained by DAPI (blue). (J) Quantification of p65 nuclear translocation by utilizing colocalization of p65 with DAPI. Data are shown as mean ± SD. **p < 0.01. Scale bar: I, *K* = 30 μm.

In order to elucidate the molecular mechanisms responsible for the anti-inflammatory properties of YQWY, we performed the Kyoto Encyclopedia of Genes and Genomes (KEGG) pathway enrichment analysis on the DEGs identified in the aortas of atherosclerotic mice. This analysis revealed that the genes down-regulated by YQWY treatment were enriched for the NF-κB signaling pathway (Figure 5(A and B)). This was confirmed by WB that YQWY reduced the degradation of IκBα and phosphorylation of p65 within the NF-κB pathway in a dose-dependent manner in the aorta of mice with atherosclerosis (Figure 5(C and D)).

The *in vitro* findings were in alignment with the *in vivo* observations. YQWY demonstrated a dose-dependent suppression of TNF-α-induced IκBα degradation and NF-κB/p65 phosphorylation in EA.hy926 cells (Figure 5(E and F)). In contrast, its inhibitory effects on the degradation of IκBα and phosphorylation of p65 in LPS-stimulated RAW.264 cells were more pronounced at high doses (Figure 5(G and H)). Phosphorylation of p65 at Ser536 is known to promote its nuclear translocation and transcriptional activation (Pradère et al. 2016). We further detected the impact of YQWY on p65 nuclear translocation using IF staining. Treatment with YQWY alone did not increase the nuclear translocation of the p65 in EA.hy926 or RAW264.7 cells. TNF-α and LPS stimulation significantly increased p65 nuclear translocation in EA.hy926 and RAW264.7 cells, respectively. Importantly, YQWY treatment significantly attenuated p65 nuclear translocation in EA.hy926 cells, and to a lesser extent, in RAW264.7 cells (Figure 5(I–K), Fig. S4A–B).

## Discussion

YQWY in traditional Chinese medicine has the effects of ‘tonifying Qi and warming Yang’, referring to strengthening vital energy and regulating immune function, as well as ‘HuoXueHuaYu’, which refers to the promotion of blood circulation to remove blood stasis (Ma et al. 2013; Song et al. 2021). This study investigated the protective effects of YQWY on atherosclerosis in the mouse model. Our findings demonstrate that YQWY markedly alleviated atherosclerosis primarily by attenuating vascular inflammation. Mechanistically, YQWY directly inhibited IκBα degradation and p65 phosphorylation in both endothelial cells and, to a lesser extent, macrophages. These findings provide direct evidence and mechanistic explanations supporting the potential therapeutic use of YQWY in managing atherosclerosis.

Despite the widespread utilization of statins for atherosclerosis treatment, their application is constrained by adverse effects such as muscle weakness or pain, rhabdomyolysis, and neurotoxicity (Bytyçi et al. 2022). This necessitates the development of more efficacious and well-tolerated therapeutic approaches. Given the pivotal role of inflammation in atherosclerosis development, targeting inflammatory pathways represents a promising strategy to treat atherosclerosis. Emerging evidence supports the clinical benefit of anti-inflammatory agents in CVDs, including therapeutic IL-1β monoclonal antibody Canakinumab (Ridker et al. 2017), and novel human anti-IL-6 ligand antibody (Pergola et al. 2021). Previous research has demonstrated that a variety of Chinese botanical drugs or plant monomers have superior pharmacological effects in the treatment of atherosclerosis. They affect various pathogenic mechanisms, encompassing reducing plasma LDL, mitigating endothelial oxidative stress, attenuating endothelial proinflammatory activation, inhibiting endothelial cell apoptosis, and decreasing platelet aggregation (Kirichenko et al. 2020). This study provides experimental evidence showing that YQWY ameliorates atherosclerosis *via* inhibiting endothelial cell activation and macrophage inflammation, further supporting the potential therapeutic applications of traditional Chinese medicine in the management of cardiovascular diseases.

The NF-κB pathway plays a key role in promoting vascular inflammation and the progression of atherosclerosis (Chen DC et al. 2023). Our findings demonstrate that YQWY effectively inhibited IκBα degradation and phosphorylation of p65, therefore suppressing the nuclear translocation of p65 in both endothelial cells and macrophages, although the effect was more pronounced in endothelial cells than macrophages. This difference could be attributed to the unique pharmacological properties of constituents in YQWY. Several components of YQWY, including astragalosides IV (Chen DC et al. 2023), salidroside (Hu et al. 2020), paeoniflorin (Chen J et al. 2018), kaempferol (Kim et al. 2012), and formononetin (Zhou et al. 2022) have been shown to possess potent endothelial-protective properties. These compounds are known to enhance nitric oxide production, reduce inflammation and oxidative stress in endothelial cells, primarily by inhibiting NF-κB pathway, which could explain the preferential effects of YQWY observed in endothelial cells. YQWY has been also demonstrated to exert anti-inflammatory effects in macrophages, although the effect appears to be less pronounced. This may be due to differences in the capacity of cellular uptake, receptor interactions, or downstream signaling pathways of various components in YQWY between the two types of cells. Further investigation is warranted to determine the mechanism underlying different cellular responsiveness to YQWY and to fully characterize its anti-inflammatory effects in different cell types involved in atherosclerosis.

## Conclusions

In summary, our study showed that YQWY exerted anti-inflammatory effects in the vasculature, including inhibition of endothelial activation and macrophage inflammation, thus protecting against the development of atherosclerosis. These actions are likely to be attributed to the inhibition of the NF-κB signaling pathway. Therefore, YQWY emerges as a promising therapeutic candidate to treat atherosclerosis in the clinic.

## Supplementary Material

Supplemental Material

Supplementary materials and reagents.docx

Supplemental tabel_20250106.docx

Supplementary cell lines.docx

## Data Availability

Data available on request from the authors. The data that support the findings of this study are available from the corresponding author, Prof. Jiang, upon reasonable request.
