# Supplementary material for: Yiqi Wenyang decoction protects against the development of atherosclerosis by inhibiting vascular inflammation
Source: Pharm Biol. 2025 Apr 20;63(1):264–74. doi: 10.1080/13880209.2025.2492650 (PMC12010649; doi:10.1080/13880209.2025.2492650)
Supplement: Supplemental Material [file IPHB_A_2492650_SM7612.docx]

**Supplemental Figure 1**


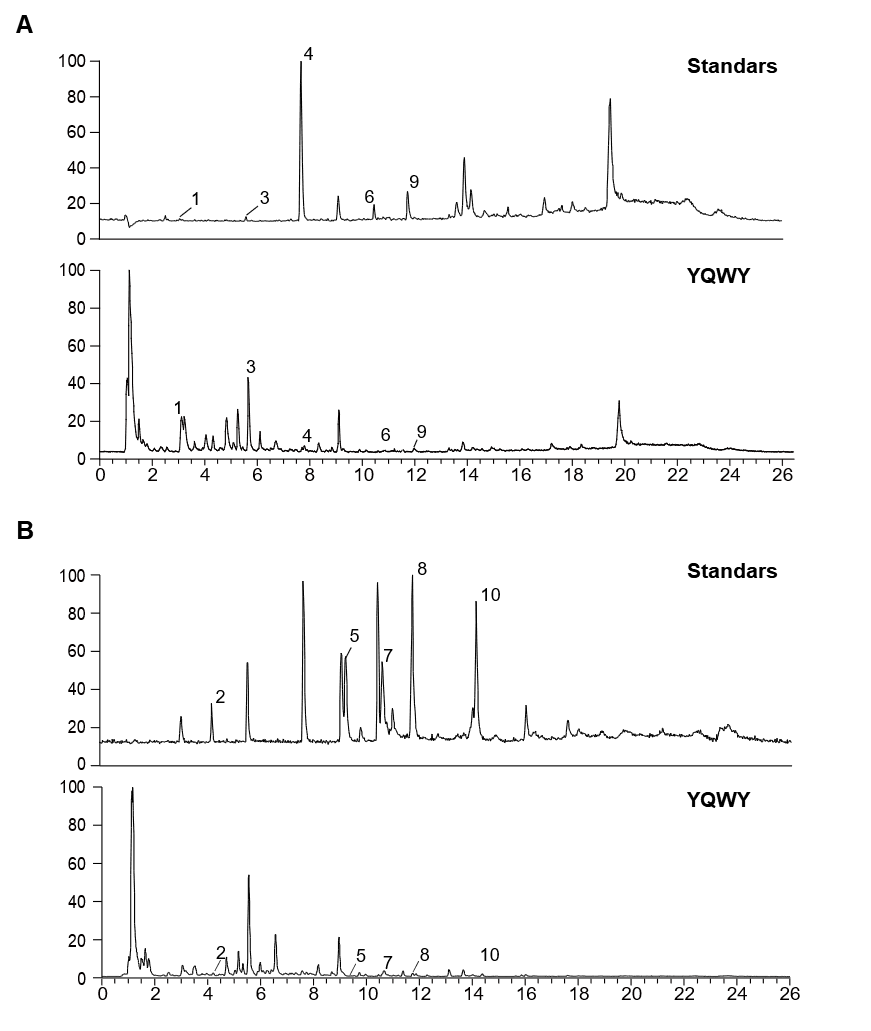


**Fig. S1: Total Ion Chromatogram of YQWY**

(A) The total ion chromatogram of YQWY in positive ion mod. (B) The total ion chromatogram of YQWY in negative ion mod. Analysis by Xcalibur 2.1SP1 software, supplemented with manual verification, identified 10 principal components and potential quality markers, such as Astragaloside IV, Salidroside, and Paeoniflorin. For detailed information on the identified components and quality markers, refer to Supplementary Table S1. LC-MS/MS was performed using an UPLC/LTQ-Orbitrap-MS mass spectrometer (Thermo Fisher Scientific). The mobile phase consisted of A: 0.1% formic acid in water B: acetonitrile and 0.1% formic acid in water. The MS detector was operated in the positive or negative ion mode with the following settings.

**
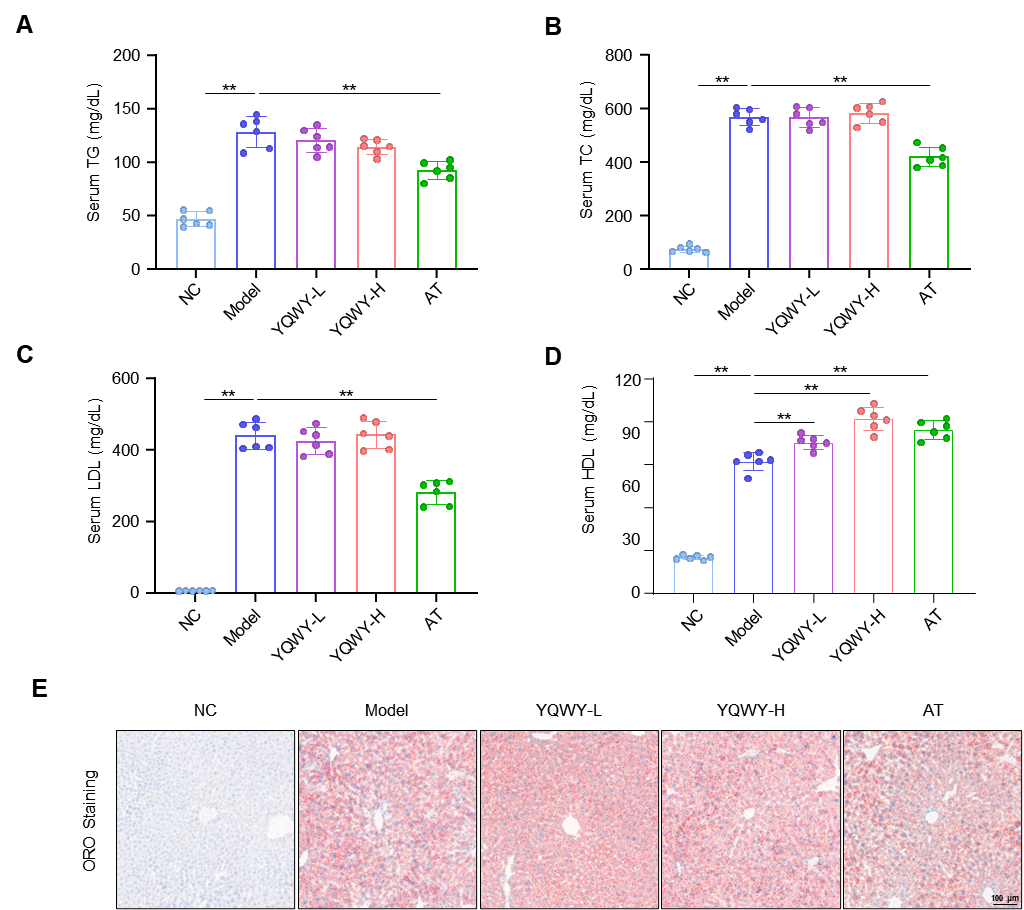
Supplemental Figure 2**

**Fig. S2: YQWY does not affect serum levels of lipids and lipoproteins in WD-fed ApoE^−/−^ mice**

8-week male C57BL/6J mice fed with STD and ApoE^−/−^ mice fed with WD were given vehicle, YQWY, and atorvastatin for 13 weeks. Fasting lipid levels in mouse serum **(A)** total triglyceride (TG), **(B)** total cholesterol (TC), **(C)** low-density lipoprotein (LDL), **(D)** high-density lipoprotein (HDL) (n = 6). All lipid values are expressed in mg/dL of plasma. **(E)** Representative Oil red O staining of liver. Data are shown as mean ± SD. ***P*  < 0.01. Scale bar: E = 100 μm.

**Supplemental Figure 3**


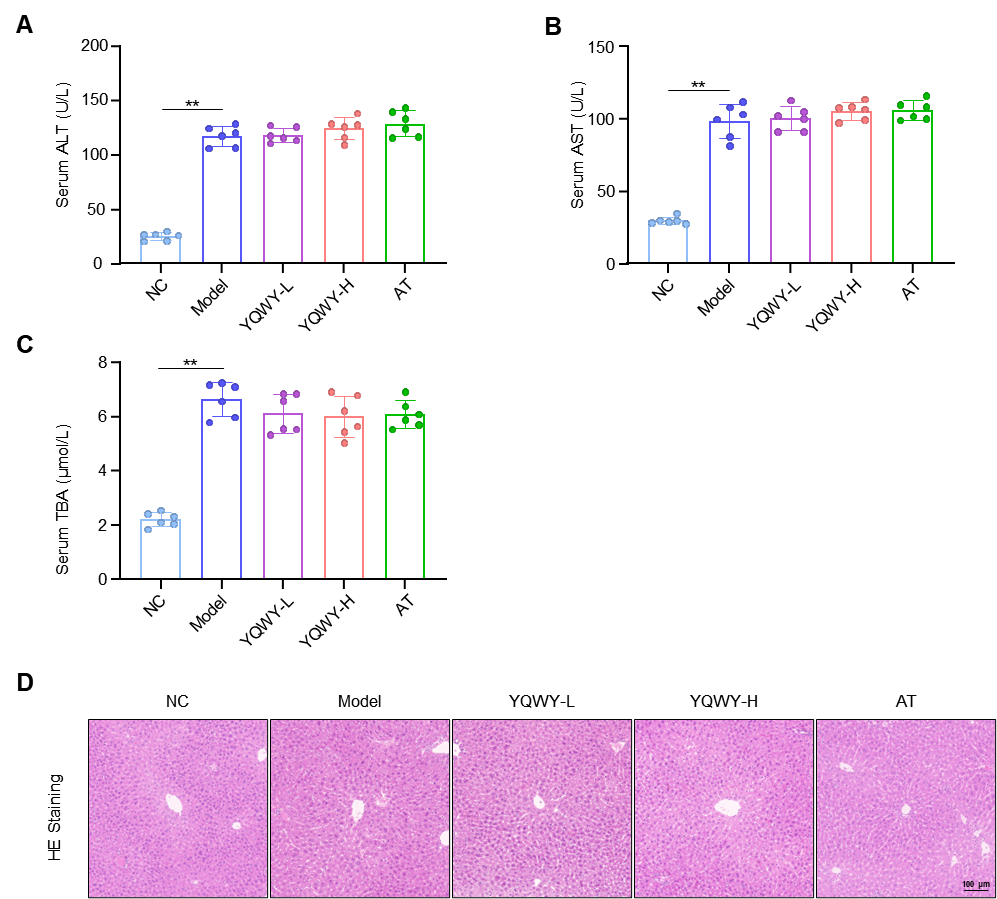


**Fig. S3: YQWY does not induce** **liver injury in WD-fed ApoE^−/−^ mice**

Vehicle, YQWY, and atorvastatin were given to 8-week male C57BL/6J mice with STD and ApoE^−/−^ mice with WD for 13 weeks. (A) Serum alanine aminotransferase (ALT) levels, (B) serum AST (aspartate transaminase) levels, and (C) total bile acid (TBA) levels (n = 6). (D) Representative photograph of HE-stained liver sections. Data are shown as mean ± SD. ***P*  < 0.01. Scale bar: D = 100 μm.

**Supplemental Figure 4**


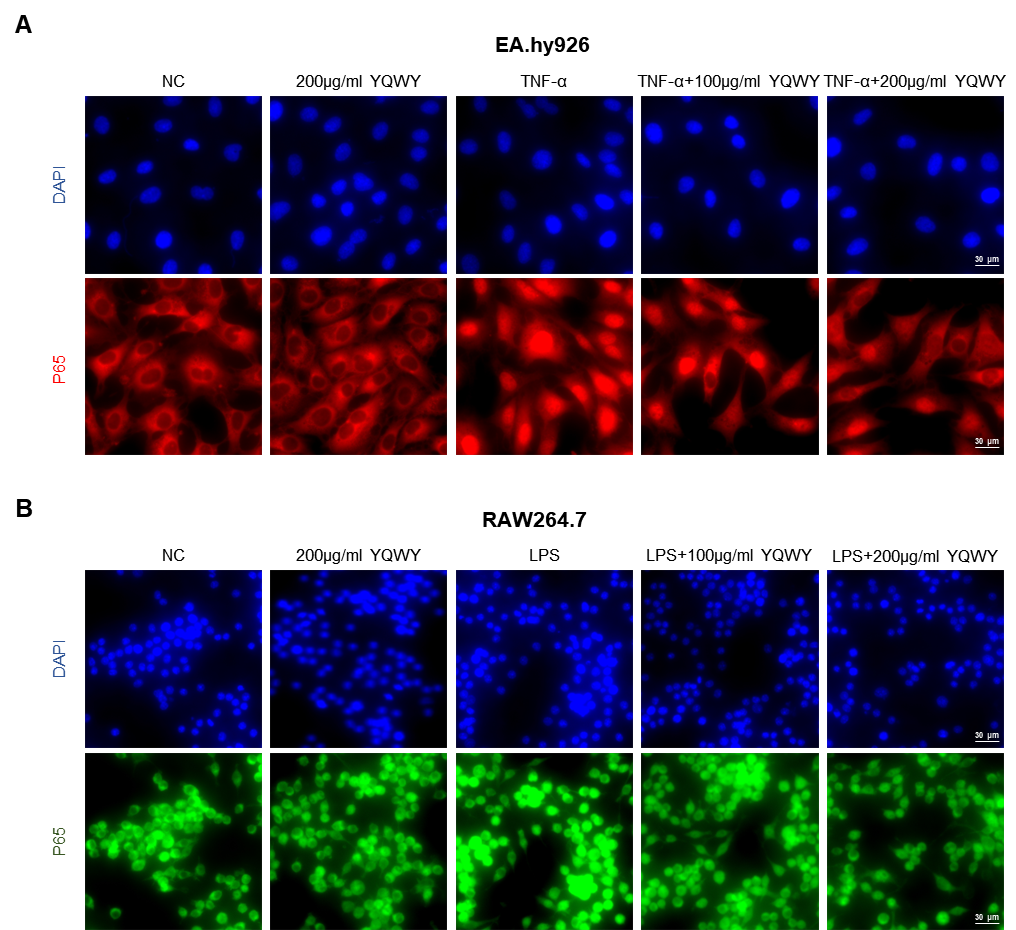


**Fig. S4: YQWY inhibits NF-κB/p65 nuclear translocation in endothelial cells and macrophages**

The EA.hy926 cells were stimulated with TNF-α and RAW264.7 cells were stimulated with LPS, and YQWY (100 or 200 μg/ml) for 30 minutes. The IF staining images of NF-κB p65 nuclear translocation in (A) EA.hy926 (red) and (B) RAW264.7 (green) cells, and nuclei were stained by DAPI (Blue). Scale bar: A-B = 30 μm.
