# Supplementary material for: Yiqi Wenyang decoction protects against the development of atherosclerosis by inhibiting vascular inflammation
Source: Pharm Biol. 2025 Apr 20;63(1):264–74. doi: 10.1080/13880209.2025.2492650 (PMC12010649; doi:10.1080/13880209.2025.2492650)
Supplement: Supplementary materials and reagents.docx [file IPHB_A_2492650_SM6845.docx]

**Supplemental materials and reagents**

| **Materials and reagents** | **Supplier** | **Catalogue numbers** |
| --- | --- | --- |
| Oil Red O | O1391 | Sigma-Aldrich |
| DAPI | G1012 | Servicebio |
| DAB | G1212 | Servicebio |
| hematoxylin | G1004 | Servicebio |
| Hematoxylin-Eosin staining (H&E) | G1005 | Servicebio |
| Masson trichrome | G1006 | Servicebio |
| Total triglycerides (TG | A110-1-1 | Nanjing Jiancheng Bioengineering Institute |
| Total cholesterol（TC） | A111-1-1 | Nanjing Jiancheng Bioengineering Institute |
| Low-density lipoprotein (LDL) | A113-1-1 | Nanjing Jiancheng Bioengineering Institute |
| High-density lipoprotein (HDL) | A112-1-1 | Nanjing Jiancheng Bioengineering Institute |
| Alanine transaminase (ALT) | C009-2-1 | Nanjing Jiancheng Bioengineering Institute |
| Aspartate aminotransferase (AST) | C010-2-1 | Nanjing Jiancheng Bioengineering Institute |
| Total bile acids (TBA) | E003-2-1 | Nanjing Jiancheng Bioengineering Institute |
| CXCL1 | AF2810-A | AiFang Biological, China |
| IL-1β | AF2040-A | AiFang Biological, China |
| TNF-α | AF2132-A | AiFang Biological, China |
| FBS | SV30208 | Hyclone |
| penicillin/streptomycin | 15140122 | Gibco |
| recombinant human TNF-α | 210-TA-020 | R&D Systems |
| LPS | L4391 | Sigma-Aldrich |
| Cell Counting Kit-8 (CCK-8) | MA0218 | Meilun |
| RNAiso Plus | 9109 | Takara |
| Reverse Transcription reaction reagent | RR037A | Takara |
| ChamQ Universal SYBR qPCR master mix | Q711-02 | Vazyme |
| RNAiso Plus | 9109 | Takara |
| Cell lysis buffer | P0013J | Beyotime Biotechnology |
| Protease and phosphatase inhibitor cocktail | P1050 | Beyotime Biotechnology |
| 10% SDS-page gel | PG112 | EpiZyme Biotechnology |
| ECL kit | SQ202L | EpiZyme Biotechnology |
