# Supplementary material for: Yiqi Wenyang decoction protects against the development of atherosclerosis by inhibiting vascular inflammation
Source: Pharm Biol. 2025 Apr 20;63(1):264–74. doi: 10.1080/13880209.2025.2492650 (PMC12010649; doi:10.1080/13880209.2025.2492650)
Supplement: Supplemental tabel_20250106.docx [file IPHB_A_2492650_SM6843.docx]

**Supplemental Table 1**. **The main chemical components of YQWY**

| NO. | Composition identification | Retention  time (min) | Detection  mode | Adduct | m/z | Molecular formula | Deviation (ppm) |
| --- | --- | --- | --- | --- | --- | --- | --- |
| 1 | Salidroside | 3.07 | Positive | [M+Na]^+^ | 323.10852 | C_14_H_20_O_7_ | -4.965 |
| 2 | Catechin | 4.21 | Negative | [M-H]^-^ | 289.07248 | C_15_H_14_O_6_ | 6.280 |
| 3 | Paeoniflorin | 5.59 | Positive | [M+Na]^+^ | 503.14932 | C_23_H_28_O_11_ | -6.087 |
| 4 | Benzoylmesaconine | 7.68 | Positive | [M+H]^+^ | 590.29218 | C_31_H_43_NO_10_ | -6.425 |
| 5 | Quercetin | 9.25 | Negative | [M-H]^-^ | 301.03595 | C_15_H_10_O_7_ | 5.551 |
| 6 | Astragaloside IV | 10.47 | Positive | [M+Na]^+^ | 807.44659 | C_41_H_68_O_14_ | -4.381 |
| 7 | Calycosin-7-O-β-D-glucoside | 10.58 | Negative | [M-H]^-^ | 445.11508 | C_22_H_22_O_10_ | 4.845 |
| 8 | Kaempferol | 10.62 | Negative | [M-H]^-^ | 285.04095 | C_15_H_10_O_6_ | 5.562 |
| 9 | Formononetin | 11.75 | Positive | [M+H]^+^ | 269.07968 | C_16_H_12_O_4_ | -4.294 |
| 10 | Curcumin | 14.14 | Negative | [M-H]^-^ | 367.11899 | C_21_H_20_O_6_ | 3.746 |

**Supplemental Table 2. Primers sequences**

| **Human** | **qPCR Primers** |
| --- | --- |
| ICAM1 | Forward: AGCGGCTGACGTGTGCAGTAAT |
|  | Reverse: TCTGAGACCTCTGGCTTCGTCA |
| VCAM1 | Forward: CTTAAAATGCCTGGGAAGATGGT |
|  | Reverse: GTAATGAGACGGAGTCACCAAT |
| CXCL1 | Forward: ATTTCTGAGGAGCCTGCAAC |
|  | Reverse: CCCTGCCTTCACAATGATCT |
| CCL5 | Forward: CCTGCTGCTTTGCCTACATTGC |
|  | Reverse: ACACACTTGGCGGTTCTTTCGG |
| ACTB | Forward: CACCATTGCAATGAGCGGTTC |
|  | Reverse: AGGTCTTTGCGGATGTCCACGT |
| **Mouse** | **qPCR Primers** |
| Icam1 | Forward: GTGATGCTCAGGTATCCATCCA |
|  | Reverse: CACAGTTCTCAAAGCACAGCG |
| Vcam1 | Forward: GCTATGAGGATGGAAGACTCTGG |
|  | Reverse: ACTTGTGCAGCCACCTGAGATC |
| Cxcl1 | Forward: ACCCAAACCGAAGTCATAGCC |
|  | Reverse: TTGTCAGAAGCCAGCGTTCA |
| Ccl5 | Forward: TGCTGCTTTGCCTACCTCTC |
|  | Reverse: TCTTCTCTGGGTTGGCACAC |
| Ccl2 | Forward: TAAAAACCTGGATCGGAACCAAA |
|  | Reverse: GCATTAGCTTCAGATTTACGGGT |
| Il1b | Forward: GAAATGCCACCTTTTGACAGTG |
|  | Reverse: TGGATGCTCTCATCAGGACAG |
| Tnf | Forward: GGTGCCTATGTCTCAGCCTCTT |
|  | Reverse: GCCATAGAACTGATGAGAGGGAG |
| Actb | Forward: GGCTGTATTCCCCTCCATCG |
|  | Reverse: CCAGTTGGTAACAATGCCATGT |

**Supplemental Table 3. List of antibody**

| **Antibodies for immunohistochemistry and immunofluorescence** | | | | |
| --- | --- | --- | --- | --- |
| Target | Company | Cat | Dilution | Clone |
| ICAM1 | Servicebio | GB11106 | 1:1000 | Polyclonal |
| VCAM1 | Servicebio | GB113498 | 1:200 | Polyclonal |
| CXCL1 | Proteintech | 12335 | 1:200 | Polyclonal |
| IL-1β | Servicebio | GB11113 | 1:2000 | Polyclonal |
| TNF-α | Proteintech | 60291 | 1:600 | 7B8A11 |
| CD68 | Proteintech | 66231 | 1:200 | 3A9A7 |
| NF-κB p65 | CST | 8242 | 1:1000 | D14E12 |
| HRP-conjugated secondary antibody anti-rabbit IgG | Servicebio | GB23303 | 1:200 | - |
| HRP-conjugated secondary antibody anti-mouse IgG | Servicebio | GB23301 | 1:200 | - |
| CY3-conjugated Goat anti-Rabbit IgG | Servicebio | GB21303 | 1:300 | - |
| Alexa Fluor 488-conjugated Goat anti-Rabbit IgG | Servicebio | GB25303 | 1:400 | - |
| **Antibodies for WB** | | | | |
| Target | Company | Cat | Dilution | Clone |
| Phospho-NF-κB p65 | CST | 3033 | 1:1000 | 93H1 |
| NF-κB p65 | CST | 8242 | 1:1000 | D14E12 |
| IκBα | CST | 4814 | 1:1000 | L35A5 |
| GAPDH | Proteintech | 60004 | 1:10000 | 1E6D9 |
| HRP-conjugated goat anti-rabbit IgG | CST | 7074 | 1:2000 | - |
| HRP-conjugated horse anti-mouse IgG | CST | 7076 | 1:2000 | - |
