# Supplementary material for: Yiqi Wenyang decoction protects against the development of atherosclerosis by inhibiting vascular inflammation
Source: Pharm Biol. 2025 Apr 20;63(1):264–74. doi: 10.1080/13880209.2025.2492650 (PMC12010649; doi:10.1080/13880209.2025.2492650)
Supplement: Supplementary cell lines.docx [file IPHB_A_2492650_SM6842.docx]

**Supplemental cell lines**

| Cell lines | Species | CVCL numbers | Catalogue numbers | Source |
| --- | --- | --- | --- | --- |
| EA.hy926 | Homo sapiens (Human) | CVCL_3901 | SCSP-5285 | the Cell Bank of Typical Culture Preservation Committee of Chinese Academy of Sciences |
| RAW264.7 | Mus musculus (Mouse) | CVCL_0493 | SCSP-5036 |  |
